# Recombination as a Way of Life: Viruses Do It Every Day

**DOI:** 10.1371/journal.pbio.0030117

**Published:** 2005-03-01

In theory, a cell's nuclear membrane guards its contents by barring access to potential foes. In reality, pathogens employ a diverse bag of tricks to circumvent this barrier. The murine leukemia virus (a retrovirus), for example, waits until the nuclear membrane degrades during cell division. Other retroviruses, like HIV and so-called pararetroviruses, enlist protein escorts that help them slip through undetected.

Pararetroviruses include both animal viruses, such as hepatitis B, and plant viruses, such as the cauliflower mosaic virus (CaMV). Once inside the nucleus, the double-stranded DNA genome of the CaMV is transcribed into an RNA transcript (called 35S RNA), thanks to the activity of the 35S promoter. (This CaMV promoter is widely used to drive transgenic expression in plants.) Replication proceeds through reverse transcription as a viral enzyme reverse transcribes the 35S RNA into genomic DNA that is then packaged into viral particles.[Fig pbio-0030117-g001]

**Figure pbio-0030117-g001:**
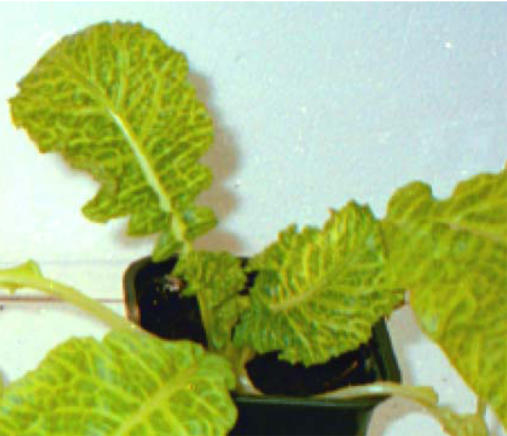
Turnip infected by cauliflower mosaic virus

During replication, genetic material can pass between different viral genomes when two viral particles infect the same host cell. These exchanges can create novel viruses, much like mutations in bacteria can produce new bacterial strains that show resistance to host defenses and antibiotics. But with little data on viral recombination rates in multicellular organisms, it's unclear how these recombinant viral genomes are influencing host infection. In a new study, Yannis Michalakis and colleagues follow the course of the cauliflower mosaic viral infection in one of its natural hosts, the turnip plant (Brassica rapa), to measure the frequency of viral recombination. Recombination was evident in over half of the recovered viral genomes, suggesting that recombination is routine for this plant virus.

It's thought that CaMV recombination occurs mostly outside the nucleus, in the host's cytoplasm, during reverse transcription. To quantify the frequency of such events, Michalakis and colleagues generated a CaMV genome with four genetic markers and then infected 24 turnip plants with equal amounts of marked and unaltered viruses. Recombination between the two “parent” genomes would produce viral populations with genetic material from both parents. The plants were harvested when full-blown symptoms developed, 21 days after inoculation, and viral DNA was extracted from their leaves to evaluate the occurrence and frequency of recombination.

Assuming that all marker-containing genomes could recombine, the authors predicted that the viruses should produce seven classes of recombinant genotypes, which is what they found. These recombinant genotypes showed up in over 50% of the viral populations—which the authors call an “astonishingly high” proportion. Though little information exists on the length of viral replication cycles in plants, the authors assumed a generation time of two days, which would amount to ten replication cycles over the 21-day experimental period. From this assumption, the authors calculated the recombination rate on the order of 4 × 10^−5^ per nucleotide base per replication cycle—hardly a rare occurrence. Certain CaMV genomic regions have been predicted as recombination hot spots, but the authors found that the virus “can exchange any portion of its genome… with an astonishingly high frequency during the course of a single host infection.”

By evaluating the recombination behavior of a virus in a living multicellular organism, Michalakis and colleagues created a realistic approximation of recombination events during infection in the field. And since recombination events are linked to both expanded viral infection and increased virulence, understanding the rate of recombination could help shed light on mechanisms underlying the evolution and pathology of a virus—insight that could prove critical for developing methods to inhibit or contain an infection.

